# Syphilis

**Published:** 1907-12

**Authors:** Henry Waldo

**Affiliations:** Consulting Physician to the Bristol Royal Infirmary.


### The Bristol

### Medico-Chirurgical Journal.

*"Scire est nescire, nisi id me*
*Scire alius sciret."*

DECEMBER, 1907.

### SYPHILIS.

The Presidential Address, delivered on October 9th, 1907, at the opening of the Thirty-fourth Session of the Bristol Medico-Chirurgical Society.

BY

Henry Waldo, M.D.,
*Consulting Physician to the Bristol Royal Infirmary.*

First of all I wish to renew my cordial thanks to you for electing me as your president. In searching for a suitable subject for an address, it occurred to me that syphilology had made great strides during recent years, chiefly owing to the discovery of the spirochæta pallida. Syphilis is truly called the imitator of very many other diseases. It is also one of the oldest, if not the oldest disease which has afflicted mankind. In Fournier's recent work on syphilis the author regards this disease as such a common cause of miscarriage, that he considers that syphilis is one of the chief factors in the causation of the present depopulation of France. I scarcely know what the staff of a hospital would find to do if syphilis in its different phases were eradicated. It offers many obscure clinical cases for investigation, and when recognised, it is one of the most satisfactory conditions to treat. One of our members told the City Council that sanitation was rapidly interfering with the gold mine of medicine. I think that if syphilitics were no more the mine might be closed down. The opinion seems to be gaining ground that syphilis is absolutely curable, excepting when it is associated with the abuse of alcohol. It was Moxon, I think, who said that spirit is the best preservative of syphilis. Its curability is borne out by the following facts.

It used to be taught that syphilis could only be acquired once in a lifetime, and that reinfection was impossible; but a number of cases have been recorded by careful observers which seem to establish the possibility of reinfection beyond a doubt. Hutchinson has observed two cases, in one of which the patient passed through two attacks of primary and secondary syphilis with an interval of five years. In the second case the patient had three attacks, with intervals of fourteen and nine years. Berkeley Hill relates the case of a surgeon who acquired syphilis in the usual way; eleven years later he contracted a digital chancre, followed by secondary syphilis. It is interesting to note that Ricord in his later years recognised the possibility of reinfection. In a letter to W. Acton in 1872, he said: "Now that we have authentic examples of fresh contagions of indurated chancres, with consecutive evolution of the whole series of constitutional symptoms, this proves that patients have been cured, just as the possibility of contracting small-pox afresh, or of vaccination again taking, proves that the first variolous or vaccine influence had ceased." Sir William Gowers has been reputed to have made the statement: "Once a syphilitic, always a syphilitic." But when he was asked to give his reasons for this at a medical meeting, he said: "I have never said such a thing, I have never thought such a thing." He believed every syphilitic ultimately ceases to be a syphilitic, and he continued: "So far from being a sceptic as to therapeutics, I would say this, that you could bring syphilis to an end in the kingdom if you kept under the influence of mercury every unmarried man between 18 and 50 years of age continuously But," he added, "you would extinguish other things besides syphilis. Promotion in the army would become mysteriously rapid, and the members of the House of Lords would gradually melt away. But," said Sir William, "I am not sure that such a price would be too high to pay for such a boon. That is," he said, "a matter of opinion. We all know Mr. Jonathan Hutchinson's view, namely that he believes that mercury is an antidote to the syphilitic poison if properly administered."

The parasitic germ of syphilis was discovered by Fritz Schaudinn two years ago (1905), and named the spirochæta pallida. It was called pallida on account of the physical properties of the germ possessing a low power of refracting light. Maclennan regards the spirochæte as only one stage in the life history of the micro-organism of syphilis. Unfortunately, a few months after his discovery Schaudinn died, at the early age of 35.

We as a profession are immensely indebted to men like Schaudinn, who devote their lives to work in their clinical laboratories, who as a rule are miserably paid, and who are not recognised in anything like the way they should be. They may console themselves in the words of Emerson, that "a man was born not for prosperity, but to suffer for the benefit of others." I am sure we all agree with the views of Mayo Robson, that there should be a closer union between the work of clinicians and pathologists. We do not make all the use we might of pathological investigation to aid our clinical observations. He thinks the deep debt that preventive medicine owes to bacteriology is likely to be equalled, or even excelled, in the realm of treatment; for not only have bacteriologists shown us how to treat infectious diseases, such as diphtheria, by antitoxic serums, but we have had demonstrated to us recently how the tissues can be rendered immune to various infective agents by inoculation, under suitable conditions that can be determined by the method of blood examination devised by Sir A. E. Wright. Robson says the chemistry of the body is as yet in its infancy, but it has a great future before it. He advocates that investigations of a private nature, not involving public health questions, should be conducted at the expense of the individual requiring them ; and in those of a more difficult and complicated character, especially when the clinical diagnosis depends on the result of the pathological findings, it seems to him desirable that the pathologist should be met in consultation. While it seemed most desirable that poor patients should not be deprived of the help afforded by pathology, yet it seemed anything but desirable that wealthy or well-to-do patients should be paying adequate fees to their medical or surgical advisers and a mere pittance to the pathologist, on whose findings may hang very vital issues.

Well, gentlemen, we are all looking forward in the hope that this important discovery of the spirochæta pallida will assist us in treating patients who may be supposed to be syphilitic. It has long been debated whether it is wise to administer mercury to a person with a primary sore, or to wait for confirmatory symptoms. Marshall says (in *Syphilology and Venereal Disease*, 1906) none of the attempts at abortive treatment by destruction or excision of the chancre have been definitely successful. The chancre has been excised a few hours after its appearance, the lymphatic glands have been removed, an abrasion has even been excised before any chancre appeared, but all these measures have failed to prevent the development of constitutional syphilis.

The failure of these abortive methods is due to the fact that syphilitic infection of the system takes place very rapidly, both by the lymphatics and blood vessels. When the chancre appears the organism is already entirely infected, or, at any rate, the infection has extended over an area too large for any form of excision to be effectual.

The recent experiments of Metchnikoff,[1] however, seem to show that syphilitic infection may be prevented by early inunction of the point of inoculation with an ointment containing from 25 per cent. to 33 per cent. of calomel. After establishing this fact by a series of experiments on monkeys, the experiment was tried on a medical student who volunteered for the purpose.

[1] *Brit. M. J.*, 1906, ii. 1510.

Syphilitic virus from two chancres was inoculated by scarifications in the balano-preputial furrow; at the same time four macacus monkeys were inoculated with the same virus. An hour after inoculation calomel ointment was rubbed into the scarifications for eight minutes in the student and in one of the monkeys. Twenty hours afterwards the same treatment was applied to another monkey. The two remaining monkeys were kept for control, and developed syphilitic chancres seventeen days after inoculation. The monkey treated twenty hours after inoculation developed a chancre on the thirty-second day. No sign of syphilis appeared in the student, nor in the monkey treated at the same time, up to three months after the experiment. As Marshall points out, these experiments may require further confirmation, yet they tend to show that syphilis may be prevented by mercurial inunction of the point of inoculation an hour after infection. But he thinks the practical application of this discovery is limited, owing to the fact that the point of inoculation is not known with certainty unless there is an abrasion. Moreover, patients seldom apply for treatment within an hour of possible infection. However, if such a case presents itself, inunction with calomel ointment at the supposed point of inoculation is indicated. Pernet says[1] that every medical man, dental surgeon and midwife, liable as they are to accidental infection, should have a pot or tube of it handy in case of need, so as to apply it with as little delay as possible. Marshall, in the following week's *Journal*, suggests that the ointment should be applied before the event.

Jonathan Hutchinson says the earlier mercurial treatment is begun the better, provided the diagnosis is certain. He also thinks that many chancres are sufficiently characterised to justify immediate treatment. We should all agree, I think, that as mercurial treatment, to be effectual, must extend over rather long periods, it is a serious and unjustifiable proceeding to commence treatment until we are certain of what we have to treat. And if the pathologist can assure us of the presence of the parasitic germ, it must be of the greatest advantage; for

[1] *Brit. M. J.*, 1907, i. 730.

although this organism has not at present been cultivated, and cannot, therefore, fulfil the postulates of Koch to prove its pathogenic nature, it has been found sufficiently frequently in syphilitic lesions to justify the assumption that it is the specific microbe of syphilis.

In the case of chancres a doubtful diagnosis may be cleared up by finding the spirochæta in the exudation. It has been found almost constantly in the primary and secondary lesions of syphilis, not only on the surface of ulcerated lesions, but also in non-ulcerated lesions, such as papules and lymphatic glands, and in the viscera. It has also been found in the blood (although rarely). Several recent observations considerably modify various previous conceptions with regard to the disease: for example, the discovery of the microbe in gummata, showing that the gumma might be contagious ; the fact that Levaditi has detected the spirochæte in the renal epithelium and various other organs in congenital syphilis, showing that the normal secretions, such as the urine, may be contagious.

The spirochæte has not been found in men or monkeys apart from syphilitic lesions. The reason why it has not been observed before in syphilitic lesions is explained by the difficulty in technique, and also by the fact that it is not always found. Metchnikoff failed to find it in 26 per cent. of the cases he examined. The London men tell me they have the very greatest difficulty in finding it. Thus a negative examination does not prove that a lesion is non-syphilitic, any more than the absence of the tubercle bacillus in lupus negatives the tuberculous nature of this affection. Levy Bing has shown that the spirochæta pallida rapidly disappears under the influence of mercury. Six or seven days after an injection of gray oil they became scarce, and a week after the second injection they had almost disappeared. Kowalewski, after demonstrating their presence, noted their disappearance after six injections of sublimate.

In the *British Medical Journal* for 16th June, 1906, Dr. W. R. Grove, of St. Ives, Huntingdonshire, gives brief histories of three cases of syphilis, presumably acquired from a baby 18 months old, with the inherited complaint. In each case the site of infection was believed to be the tonsil. A leading article appeared in the same journal saying that the view that the power of infection in inherited syphilis was much exaggerated by writers in the past is very widely held nowadays, and discrediting the belief that inherited syphilis is infectious. At any rate, in the light of more recent knowledge, the editor thought many would now consider the evidence as insufficient. In the following week's *Journal* a letter appears from Mr. Jonathan Hutchinson, in which he says that he has read Dr. Grove's communication with much interest, and also the editor's comments on it not without amazement. He had supposed that the contagiousness of inherited syphilis in infants was a fact about which no controversy was possible. He said it was, as all knew, in order to explain cases in which exposure to risk occurred without ill result that Colles formulated the law now known by his name.

Mr. Hutchinson says he is quite in the dark as to what the "more recent knowledge" referred to may be. The only more recent knowledge with which he is acquainted is that which concerns the spirochæte, and this he considers is entirely confirmatory of his conclusions, as this parasite has been found repeatedly in the early lesions of inherited syphilis, and he says that as it is now held to be the efficient agent in contagion, it is difficult not to consider its presence as proof that this form of the malady may be communicable. Primary infection of the tonsil in connection with the feeding of this class of infants has been witnessed repeatedly. Mr. Hutchinson reminds his readers that not so very long ago one of our associates had to pay a considerable sum to a wet-nurse whom he had inadvertently permitted to suckle an infant suffering from syphilis, which was believed to have been inherited. He concludes by saying that he must hold that to propound unsupported doubts involves not only a very foolish heresy, but a very dangerous one; and he much regrets that it should seem to have received in any degree the support of the editorial pen of the *British Medical Journal.*

Some years ago I was asked by a lady to attend her in her first confinement. This I was obliged to decline, as there is a rule at our Infirmary prohibiting a physician from practising midwifery, and so the patient was passed on to a medical friend. Some little time after the birth of the child the mother consulted me for an eruption on her fingers which much resembled scabies, although it did not itch. After a little interrogation as to whether she was able to suckle her baby she said, "Oh, yes!" but that it was rather painful on account of the nipple being sore. After examining this sore, I came to the conclusion that it was of the nature of a chancre, and that the child was the subject of congenital syphilis. They were both very amenable to treatment, but after a time the child developed a large liver, in all probability lardaceous disease, and died with dropsical symptoms and other signs of congenital syphilis. Well, this case was clearly an exception to what is known as Colles's law, and I have often intended to publish it. It was in 1837 that Colles, of Dublin, pointed out that the mother of a syphilitic infant, procreated by a syphilitic father, was herself immune against infection, and could suckle her child with impunity; while a healthy wet-nurse could contract a chancre of the nipple by suckling the same child. Since this time other well-authenticated cases of exception to this law have been recorded, where mothers contracted chancres of the nipple by suckling their syphilitic infants. Pernet says[1] that Colles's law is right; and that Colles's law is a law I have myself no doubt whatever. The alleged exceptions, he says, when examined, cannot hold water. It is obvious, therefore, he continues, that the mother should also be treated, especially from the point of future pregnancies.

Upon inquiring into the history of the father in my case, I found that he contracted syphilis in the usual way some years before marriage, that the treatment he received was a mere farce, and that he was addicted to alcohol, which has been well called the born enemy of syphilitics.

When may a syphilitic safely marry is a question which is not easily answered. Mr. Hutchinson differs from most other authorities in stating (as recently as June, 1906) that if the treatment has been continued for two years from the date of

[1] *Brit. M. J.*, 1907, i. 734.

the chancre a man may safely marry, but that a much longer period is necessary for a woman. He also remarked: "I have let hundreds of such patients marry, and they have never come back to me in consequence of having infected their wives, or having syphilitic children." Marshall thinks that this statement cannot be accepted as evidence of the safety of the two years' system, for, in case of failure, it is probable that the patients would not come back. In this way, he adds, the successes are recorded, but the failures unknown.

Fournier lays down the law that no syphilitic should marry for at least three or four years after the onset of the disease, and then only when the following conditions are fulfilled: (1) absence of actual lesions; (2) prolonged treatment; (3) a period of immunity from symptoms for at least one and a half to two years, during which time no treatment has been taken; (4) a benign type of disease. Fournier relates the history of twenty cases of marriage in syphilitics under two years; nineteen of the wives were infected, and there were twenty-eight pregnancies, resulting in thirteen abortions, six early deaths, six syphilitic infants, and only three healthy children.

Ledermann says: "Generally speaking, marriage may be allowed if at least five years have elapsed since the infection took place, if no more manifestations have occurred during the last two years, and if the patient has received an energetic and thorough mercurial treatment."

Baltzer is of opinion that marriage may be allowed during the fifth year, provided that the symptoms have been benign, the treatment regularly followed, and that there have been no symptoms during the fourth year. He also advises a course of preventive treatment immediately before marriage, and during the year following. In the case of a syphilitic woman, he says, preventive treatment is required for a long time, because the power of maternal transmission lasts much longer than that of paternal heredity.

Marshall thinks that any arbitrary time-limit is both dangerous and unscientific.

In referring to later forms of syphilis, a subject which resolves itself into syphilitic disease of the arterial system, Darier says: "To study syphilitic arteritis in its widest sense is to deal with the whole pathology of syphilis." How many cases of so-called cerebral hemorrhage, heart failure and the like are due to syphilis. Arterial disease especially affects the arteries of the brain and of the heart—possibly of the spinal cord—in the earlier period, and accounts for general paralysis and tabes dorsalis in the later period.

Ferrier suggests that the terms cerebral tabes, spinal tabes, and cerebro-spinal tabes should be substituted for general paralysis, locomotor ataxy, and tabo-paralysis.

Sir William Gowers says hemiplegia from specific arterial disease is a malady in which we can make a confident pathological diagnosis from clinical facts. Every case, he says, of sudden hemiplegia—sudden, and therefore of vascular origin—occurring between the ages of 18 and 48, without Bright's disease, without heart disease, in an individual who is known to have had syphilis, may be confidently ascribed to syphilitic arterial disease. He has been through the facts of forty cases of that kind, all conforming to those conditions, and he finds that about one quarter of them occur in the first two years after acquiring syphilis, about one half in the first five years, nearly three-quarters in the first seven years, four-fifths in the first ten years, and the others are scattered over the next seven years. And as regards surgical maladies, Marshall points out that syphilitic arteritis affecting the vessels of the lower limbs may lead to gangrene, and cases of so-called "senile gangrene," occurring in middle-aged subjects, are now recognised by surgeons as usually due to syphilis. Aneurism of the lower limbs, too, is frequently due to the same cause. Or, again, effusion of fluid into a knee-joint, with sub-acute symptoms only, is often syphilitic. It differs from common rheumatism in only affecting one or two joints, and exclusively the larger ones such as the knee, and in the absence or transient nature of febrile symptoms. In gonorrhœal arthritis the affection is more acute and painful, and there is usually posterior urethritis present.

It is astonishing how authorities differ in regard to the prognosis of late forms of syphilis. Pye-Smith says: "I confess I take a somewhat more favourable view of the outlook in cases of tabes than Sir William Gowers does. Perhaps," he says, "it is due to a smaller experience, but a few cases in which the result has been favourable make a great impression upon one; and while agreeing with him," he adds, as I am afraid all of us must as to the hopelessness of general paralysis, "I think cases of tabes are much less formidable. They are not so common, they last very much longer, often with long intervals of improvement or, at all events, abatement of the disease, before it is ultimately fatal. And sometimes—perhaps," he says, "it shows too sanguine a temperament—I have seen what looked like favourable results from mercurial treatment in tabes, whereas I do not think anybody has found such a result in general paralysis."

Jonathan Hutchinson says (at almost the same time, 1906): "Degenerative changes, such as tabes, are rarely arrested by specifics. All conditions attended by inflammatory changes, such as general paralysis of the insane, should be treated by small doses of mercury continued permanently." In his belief, if treatment of general paralysis be commenced in the very early stage a cure may be obtained, but small doses of mercury must be permanently continued. I have seen it stated that if some of the inmates of lunatic asylums had blue ointment regularly rubbed into their scalps there would be surprising results.

Dr. G. H. Savage has somewhat recently pointed out[1] that in cases of general paralysis of the insane the memory was not markedly affected. "Sometimes," he says, "as in advanced stages of general paralysis, there was a quite remarkable retention of memory, whilst in other forms of progressive mental decay, associated with alcoholism or senility, the loss of memory was the chief characteristic." Dr. Savage has also mentioned that among certain races—the Arabs and perhaps the Japanese—syphilis may be frequent and general paralysis rare. He adds that general paralysis was also said to be unknown amongst

[1] *Brit. M. J.*, 1906, i. 1344.

negroes and amongst the Irish. In his opinion syphilis required something more in order to produce this disease—alcoholism, worry and the stress of life, and perhaps large meat eating.

Widal has drawn attention to the fact that both in tabes and general paralysis leucocytosis of the cerebro-spinal fluid was constant and extremely well marked, the average being one hundred and thirty in the field. He thinks the presence of this leucocytosis is of great diagnostic importance; for example, as between tabes and peripheral neuritis, and between general paralysis and epilepsy.

However, apart from general paralysis, it has been stated that syphilis may cause all the common forms of insanity, from the most violent mania to the most complete idiocy, and, as Mott remarks, "Of all the causes of insanity, none writes with such a broad and indelible hand as syphilis." Barker says: "Considering the predilection of syphilis for the nervous system, it is remarkable that insanity is not more widespread than it is."

Some few years ago I was consulted by a male patient between 50 and 60 years of age for a sore throat. The tonsils were red and swollen with the appearance of "snail track," which is so suggestive of syphilis. There was also an asymmetrical palmar psoriasis with affection of the nails. After taking mercury for some time I sent him to see Mr. Hutchinson, who advised me to continue with the same treatment for six months. This advice was carried out very irregularly, and in addition to this drawback the patient was addicted to the alcoholic habit. After this he rather suddenly developed delusional insanity, was most restless and difficult to manage. I took ordinary precautions, and put him on gradually increasing doses of iodide, and rubbed in five per cent. oleate of mercury. Later on his relatives thought that he should be certified, but I could see that he was improving, and he very soon made a complete recovery. If I had not happened to know that this patient was syphilitic I do not think it would have occurred to me that he was the subject of syphilitic insanity. Whether this was a case of early general paralysis cut short by treatment I am not sure.

One day a male patient about 30 years of age came to see me on his bicycle. He was exceedingly breathless, and could scarcely speak. Upon examining his chest I discovered that he had an aortic aneurism, and that one of his vocal cords was not acting owing to pressure on the recurrent laryngeal nerve. I advised him to go home to bed, and wait there till I came on the following day. I induced him to stay in bed for about three months, during the whole of which time he was taking rather large doses of iodide, and the improvement was very marked. About this time his children were being attended by their family doctor, and he asked this gentleman to examine his aneurism, and the opinion given was that he could not detect an aneurism at all. This doctor, however, suggested that Dr. Michell Clarke should meet him in consultation, and then the aneurism was found to be very much in evidence. After this the patient put himself under the care of an American doctor, who said that he quickly cured aneurism, and induced the patient to believe that he had cured him. The patient then wrote to one of the Bristol daily papers abusing Dr. Clarke and myself, and pointing out how very much English doctors were behind their American brethren. Some time after this Dr. Cory asked me to see this same patient with him, and I found him in a condition of acute mania. I thought that he would put his fist through the window pane every moment. We at once certified him, and he went to Fishponds Asylum, and I think it was through the zeal of Mr. A. L. Flemming that the aneurismal specimen found its way into our museum at the Infirmary. I have often regretted that I did not include mercury in the treatment when I had him in bed; perhaps the insanity might have been thereby avoided.

In all these nervous affections there is a simple and reliable sign which is worth special attention in making a differential diagnosis. It is that the loss of pupillary reflex to light constitutes by itself a sign which is almost pathognomonic of a syphilitic lesion of the nervous centres: the pupil is fixed.

Thus the Argyll-Robertson pupil is not the only pupillary sign of syphilis. For this help in diagnosis we are indebted to Babinski and Charpentier.

Syphilis sometimes attacks the aorta, and prepares it for aneurismal bulging. It less often limits its attentions to the aortic valves. Hale White says[1] : " As for valvular disease, the aortic valves were rarely affected alone in rheumatism. Therefore, cases of aortic without mitral disease might be attributed to atheroma, or strain, or syphilis ; and in women, as strain was rare, aortic disease was strong evidence of syphilis. The same applied to aneurism."

Some few years ago Dr. Shingleton Smith asked me to see a young woman in one of his wards at the Infirmary with symptoms somewhat resembling those of rheumatic fever. There was a loud murmur over the base of the heart, the patient was acutely ill, and seemed likely to do badly. Her symptoms did not respond to salicylates. There was an eruption over the body which was really the key to the situation. The condition had already been recognised by Dr. Smith, and the aortitis, with the other manifestations of syphilis, gave way to specific remedies, the patient made a good recovery, and I think lost all signs of the cardiac trouble, I may safely say, through the means of a skilful diagnosis.

As regards eruptions on the skin, Norman Meachen thinks that by cultivating the sense of touch it is possible to recognise whether the rash is syphilitic or not. There is he says a good deal of infiltration in a syphilitic eruption, and they all, except the macular, give one the impression as if the lesions were let into the skin, the whole structure of which seems to participate in the morbid process, and not the epidermis alone as in psoriasis, eczema, and most others.

The most inveterate case of syphilis I ever saw was in a woman who attended pretty regularly at the Infirmary for fifteen years. The disease expended itself chiefly on the face and neck, and left extensive cicatrices. There was a good deal of the intradermic form of lenticular syphilide in this case, which forms the atrophic papular syphilide by destruction of the elastic tissue of the dermis, causing depressions simulating lineæ albicantes.

The eruption in this case was always controlled by iodides, but believing as I do that the disease is only scotched by these remedies, and not killed, she consumed a large quantity of mercury besides. I do not think she was an alcoholic, but she was badly fed, and so carried out my instructions of taking a quart of milk daily. She was also told to be in the open air as much as possible, and to sleep with an open window. Latterly I adopted the plan of intra-muscular injections of mercury, and then handed her over to Dr. Nixon, who, I believe, continued this mode of treatment. Hutchinson says intra-muscular injections are very dangerous, excepting in the hands of the expert, and should be wholly reserved for special conditions, chiefly in the army. If salivation commences, he says, it cannot be stopped except by excision of the portion of muscle containing the mercury.

This woman's husband became insane, and an inmate of the Fishponds Asylum ; probably a subject of syphilitic insanity.

Barker thinks that the cause of malignant syphilis is doubtful ; it has been attributed both to excessive virulence of the microbe, and to secondary infection with other organisms. It would appear, he says, more probable that it is due to the implantation of the microbe on virgin soil, *i.e.* on persons whose ancestors have been free from syphilis.

Whether the treatment of syphilis shall be continuous as advocated by Hutchinson, or intermittent as carried out by Fournier, is a matter of opinion. No doubt that for a time mercury increases the hæmoglobin and the red corpuscles : later on it diminishes them. This indicates that mercury should only be given for short periods at a time, and lends support to "the chronic intermittent treatment of Fournier."

There are many other diseased conditions which result from the parasitic germ of syphilis, and more especially when it is untreated in its early stages, often cases which are known as Lues insontium and syphilis ignota. Byrom Bramwell remarks : "Why is it that one man who drinks gets cirrhosis of the liver, while another man who drinks does not ? Is it that the man who gets cirrhosis of the liver has had syphilis?" Or as he says in other words, syphilis renders the connective and vascular tissues of the liver more vulnerable to other exciting causes.

It is thought by some good authorities that syphilis is the cause of Bright's disease. Huchard says: "Interstitial nephritis, before becoming a disease of the kidney, is an affection of the cardio-arterial system."

Barker thinks that as syphilis is essentially a disease which affects the vascular system, it is rational to regard it as an important factor in the causation of Bright's disease.

Professor Poirier says[1]: "Everybody cannot have cancer of the tongue; two conditions are almost indispensable—you must be a smoker or syphilitic; and those who combine those two conditions, especially the latter, run a much greater risk than other people. Cancer of the tongue," he says, "might be called the cancer of syphilitic smokers."

Professor Fournier[2] finds that among one hundred and eighty-four cases of cancer of the mouth seen in his private practice, one hundred and fifty-five had decided syphilitic antecedents, a proportion of at least eighty-four per cent. In the twenty-nine other cases the antecedents were not given.

It has been suggested by Pernet that the spirochæta pallida swarms at night, and so may account for the nocturnal pains of syphilis. In some cases of neuritis, and especially in sciatica, if the pain is worse at night it is usually best relieved, I think, by antisyphilitic remedies.

In conclusion, gentlemen, I should like to say that no one is more conscious than myself of the shortcomings of this paper, and I sincerely thank you for your kindness, attention and patience. In the words of Faust:—

> "Expression, graceful utterance is the first and best acquirement of the orator. This do I feel, and feel my want of it."

NOTE.—Since the above address was written the cultivation of the spirochætes has been carried out in the rabbit's eye through five generations, and finally inoculation into the monkey has produced syphilis.[3]

[1] *Brit. M. J.*, 1906, ii. 1668. [2] *Ibid.*

[3] Bertarelli, *Zentralb. für Bakt.*, 1906-7.

